# The Impact of p53 Dysfunction in ATR Inhibitor Cytotoxicity and Chemo- and Radiosensitisation

**DOI:** 10.3390/cancers10080275

**Published:** 2018-08-20

**Authors:** Fiona K. Middleton, John R. Pollard, Nicola J. Curtin

**Affiliations:** 1 Northern Institute for Cancer Research, Newcastle University, Newcastle upon Tyne NE2 4HH, UK; fmiddleton@cancertechnology.com; 2 Vertex Pharmaceuticals (Europe) Limited, Milton Park, Abingdon, Oxfordshire OX14 4RY, UK; John_Pollard@vrtx.com

**Keywords:** ATR, p53, radiation, gemcitabine, cytotoxicity, cell cycle

## Abstract

Ataxia telangiectasia mutated and Rad3 related kinase (ATR) signals replication stress and DNA damage to S and G2 arrest and promotes DNA repair. Mutations in p53, critical for G1 checkpoint control, are common in cancer and predicted to confer vulnerability to ATR inhibitors. Reported data on the impact of p53 status are variable possibly because of the use of unmatched cells and surrogate endpoints of survival. The cytotoxicity of VE-821 alone and its ability to potentiate radiation and gemcitabine cytotoxicity was determined in isogenic and unmatched p53 wild-type (wt) and null/mutant cells, as well as immortalised nonmalignant MCF10 (immortalised non-neoplastic) cells, by colony-forming assay. The effect on cell cycle checkpoints was determined by flow cytometry. The isogenic p53 defective cells were not more sensitive to VE-821 alone. Defective p53 consistently conferred greater chemo- and radiosensitisation, particularly at high dose levels in isogenic cells but not unmatched cells. VE-821 did not sensitise MCF10 cells. We conclude that p53 status is just one factor contributing to chemo- and radiosensitisation by ATR inhibition, the lack of chemo- or radiosensitisation in the noncancerous cells suggests an element of tumour-specificity that warrants further investigation. The greater sensitisation at high-dose irradiation suggests that ATR inhibitors may be most effective with hypofractionated radiotherapy.

## 1. Background

The DNA damage response (DDR) is essential for survival in the face of the high level of endogenous and environmental DNA damage sustained on a continuous basis. It comprises a highly orchestrated network of proteins that identify DNA damage, signal it for repair and cell cycle checkpoints to prevent damage being carried through to the next generation. One of the most versatile elements of the DDR is the Ataxia-telangiectasia-mutated and Rad3-related kinase (ATR). ATR is activated by single-stranded DNA (ssDNA) that primarily arises as a result of replication stress, but also following the resection of DNA double-strand breaks (DSB) and nucleotide excision repair intermediates. Replication stress can occur due to the replication fork meeting a DNA lesion or replication rate outstripping nucleotide supply e.g., through activation of proliferation-driving oncogenes such as MYC and RAS or reduced nucleotide biosynthesis following antimetabolite therapy. ATR’s primary downstream target is CHK1, which signals to S and G2 arrest and both ATR and CHK1 help to recruit and activate components of homologous recombination DNA repair (HRR) (reviewed in [1]).

Inhibiting the DDR has the potential for tumour-selective cytotoxicity when it exploits a tumour-specific DDR defect. This potential for synthetic lethality is an exciting new concept in cancer therapy, exemplified by the cytotoxic activity of PARP inhibitors in cancers with defective HRR, proving effective in the clinic [2]. The tumour suppressor p53 plays a crucial role in the DDR, principally signalling DNA damage to the G1 cell cycle checkpoint. Mutation or loss of p53 is estimated to occur in around 50% of cancers [3]; therefore, exploiting this defect with targeted anticancer therapy has significant potential. Cells harbouring a p53 mutation are likely to have more replication stress and also be more dependent on S and G2/M checkpoint control. Both ATR and CHK1 are therefore highly attractive targets for cancer therapy, not only to target cancer cells with oncogene-induced replication stress, but also to exploit the high level of p53 defects in cancer [4]. Consistent with this, it has been shown in a range of preclinical models that inhibitors of ATR and CHK1 have cancer-specific activity when used in combination with DNA-damaging chemotherapy. Much of the rationale for the development of CHK1 and ATR inhibitors has been to exploit tumour-specific defects in G1 control due to p53 defects.

There is substantial evidence for chemosensitisation by ATR inhibitors preclinically in p53 mutant or null cells (reviewed in [1]). However, only a few studies have directly compared the effects of ATR/CHK1 inhibitors in p53 wild-type (wt) and dysfunctional cells (reviewed in [5]). Experiments in isogenic HCT116 p53 null and wt cells revealed that ATR knockdown or mutation (ATR^S/S^) enhances cisplatin cytotoxicity in a p53-dependent manner [6]. Similarly, in isogenic cell lines ATR inhibitors potentiated cisplatin to a greater extent in cells where p53 had been knocked down or degraded [7]. Most recently, in a panel of 29 primary CLL samples, TP53 defective (*n* = 6) samples were substantially more sensitive to ATR inhibition than wild-type cells [8]. However, it is important to acknowledge that other studies have shown that p53 competent cancer cells can also be sensitive to ATR inhibitors [9,10]. It is clear that oncogenic stress, e.g., due to Myc or Ras amplification and defects in other components of the DDR, particularly ATM, are also synthetically lethal with ATR inhibition or confer increased sensitivity to ATR chemosensitisation [1,11].

Four ATR inhibitors are undergoing clinical trials, VX-970 (M6620, similar to VE-821), M4344 (VX-803), AZD6738, and BAY1895344 as single agents and in combination with gemcitabine, platinum agents, topoisomerase I poisons and radiotherapy (clinicaltrials.gov). Although p53 status is easily determined, it is not clear if it is a useful biomarker for patient stratification for ATR inhibitor therapy. The aim of the study reported here was to assess how much of a determinant of sensitivity p53 is using matched colon cancer and osteosarcoma cells and unpaired breast cancer cells. We used the ATR inhibitor VE-821 to enable a direct comparison with our previously published data with this ATR inhibitor [11,12], and a study in p53 wt and mutant cell lines [7]. Whilst the potency and pharmacological properties of VE-821 preclude its clinical development, it is an ideal tool for determining the effect of ATR inhibition preclinically. We report that p53 status is not a significant determinant of sensitivity to VE-821 as a single agent. However, in the matched cell lines VE-821 sensitised the p53 defective cells to gemcitabine and irradiation to a greater extent than the wt cells. This could not be attributed to p53-specific effects on the cell cycle. In the unmatched breast cancer cell lines, gemcitabine sensitisation was greater in the p53 mutant MDA-MB231 cells than MCF7 cells but radiosensitisation was similar. Reassuringly, VE-821 did not have a significant effect on the cytotoxicity of gemcitabine or radiation in immortalised human nontumourigenic breast epithelial MCF10A cells.

## 2. Results

### 2.1. VE-821 Inhibits ATR Activity on All Cell Lines

**Figure 1:**
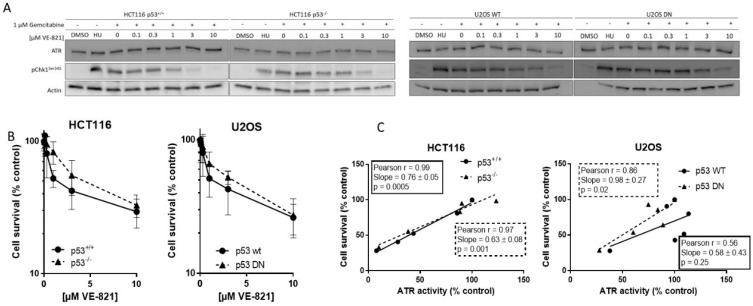
Activity of VE-821 as a single agent.

We measured ATR activity by CHK1 phosphorylation at serine 345, as this was the most specific indicator of ATR activity determined in our previous studies [9,12]. Hydroxyurea was used as a positive control and equivalent ATR activity was induced following exposure to gemcitabine. Co-exposure to VE-821 caused a concentration-dependent decrease in CHK1 phosphorylation in all cell lines. Figure 1A shows representative blot from matched HCT116 and U2OS cells. The concentration of VE-821 needed to inhibit CHK1 phosphorylation by 50% (IC_50_) varied between the cell lines (Supplementary Table S1) but was not related to the p53 status and may have been influenced by the inherent difficulty in quantifying Western blots and interassay variation.

### 2.2. Cytotoxicity of Single-Agent VE-821 Is Not Greater in p53 Mutant Cells and Cytotoxicity Is Directly Proportional to ATR Inhibition

To determine if basal endogenous levels of replication stress were sufficient to require cell cycle checkpoint function and greater dependence on ATR signaling in cells with p53-dependent GI checkpoint dysfunction, we exposed the matched pairs of cells to VE-821 alone. VE-821 was cytotoxic to HCT116 p53^+/+^ and p53^−/−^ cells and U2OS p53 wt and dominant negative mutants cells (Figure 1B). Neither of the p53 dysfunctional cells were more sensitive than their wt counterparts. In fact, they appeared to be marginally more resistant, although there was no significant difference in the LC_50_ concentration (Supplementary Table S2). Similarly, as we have reported previously [13], the concentration of VE-821 required to kill 50% of MCF7 and MDA-MB-231 cells was similar and the p53 status was not a determinant of sensitivity. Importantly, while the LC_50_ values in the cancer cells were in the range 2–5 µM, VE-821 was minimally cytotoxic in noncancerous MCF10A breast cells where survival was >50% at the highest concentration used; 10 µM.

To determine if survival was related to residual ATR activity remaining after exposure to VE-821 the percentage of cell that survived was compared with the percentage ATR activity (pChk1^Ser345^) remaining, relative to untreated control, following exposure to the same concentrations of VE-821. There was a clear and significant strongly positive correlation in both HCT116 cell lines and in the U2OS cells with mutant p53 (Figure 1C), indicating that survival is dependent on ATR activity independently of p53 status.

*Target inhibition*: Representative Western blot using lysates of cells treated with DMSO alone (control), hydroxyurea (HU; positive control) gemcitabine (1 µM) alone and in combination with the indicated concentrations of VE-821 for 1 h (A). + denotes addition of gemcitabine.

*Single-agent cytotoxicity*: Cells were exposed to increasing concentrations of VE-821 for 24 h prior to reseeding for colony formation (B). Survival curves generated from data pooled from ≥3 individual experiments are shown.

Correlation of cell survival at each concentration of VE-821 with remaining ATR activity at the same VE-821 concentration (C).

### 2.3. ATR Inhibition Potentiates Gemcitabine, in p53 Dysfunctional and wt Cells, but Not Nontumourigenic Immortalised Breast Cells

**Figure 2:**
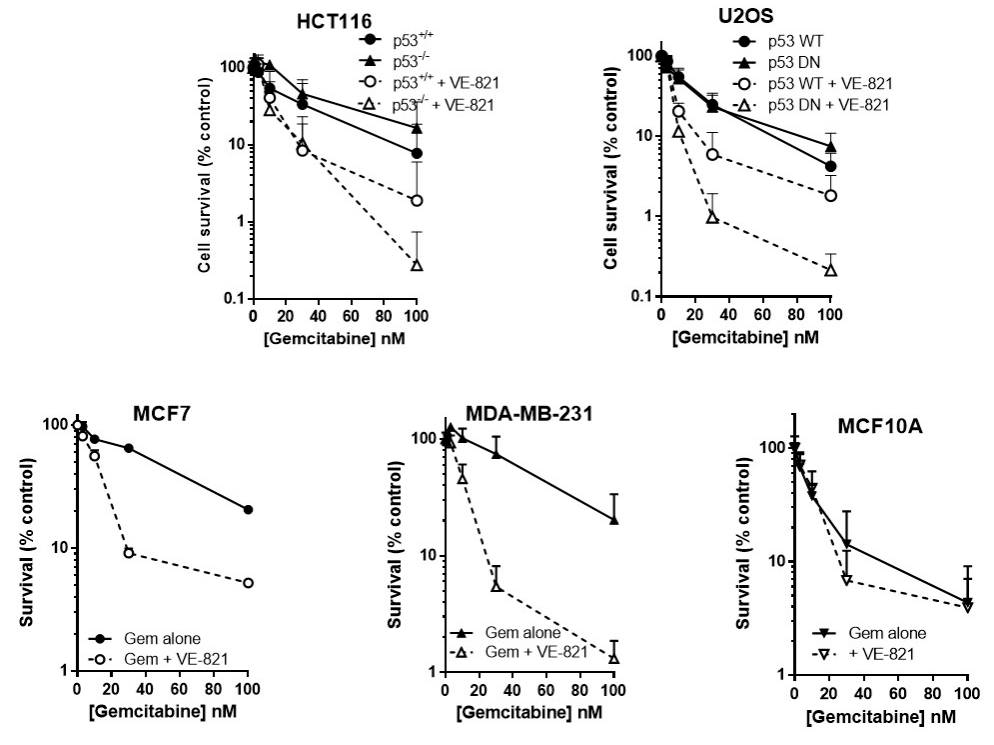
VE821 potentiates gemcitabine, irrespective of p53 status, but not nontumourigenic immortalised breast cells. Cells were seeded into 6-well plates and allowed to adhere for 24 h. Following 24 h co-exposure to gemcitabine alone (filled circles, solid line) or gemcitabine + 1 µM VE-821 (open circles, dashed line) cells were counted and reseeded at low density for colony formation. Colonies were allowed to grow for 2 weeks before being fixed, stained, and counted. Data are the mean + SEM of 3 individual experiments.

As predicted, VE-821 potentiated gemcitabine cytotoxicity to a greater extent in cells with either deleted or mutated p53 than those with wild-type p53 (Figure 2). In p53^−/−^ HCT116 cells, VE-821 caused a 4-fold reduction in the LC_50_ value (Supplementary Table S3) that was significant (paired *t*-test, *p* = 0.035) but only a 1.5-fold potentiation of the p53 wt cells that was not significant. VE-821 significantly potentiated gemcitabine in both p53 wt and p53 dominant negative mutant (DN) U2OS cells (2-way ANOVA, *p* < 0.05). Potentiation was modest at concentrations at or below the LC_50_, and similar in wt and DN p53 U2OS cells. However, at 100 nM gemcitabine VE-821 caused a significantly greater sensitisation in the p53 dysfunctional cells (60-fold for HCT116 p53^−/−^ and 35-fold for U2OS p53DN) compared to their wt cells (4.1-fold for HCT116 and 2.3-fold for U2OS).

VE-821 also significantly potentiated gemcitabine in MCF7 cells (2-way ANOVA, *p* < 0.0001) and MDA-MB-231 cells (2-way ANOVA, *p* < 0.0001) (Figure 2). Furthermore, VE-821 reduced the gemcitabine LC_50_ more than 4-fold in MCF7 cells and MDA-MB-231 cells (Supplementary Table S3). Again, at 100 nM gemcitabine there was a striking difference in the chemosensitisation, which was 15.5-fold for the p53 mutant MDA-MB-231 cells compared with only 3.9-fold in the p53 wild-type MCF7 cells. In marked contrast, there was no potentiation of MCF10A cells (Figure 2, 2-way ANOVA, *p* = 0.87) and no reduction in LC_50_ values (Supplementary Table S3). Based on these limited data, there is some suggestion that the potentiation of gemcitabine may be both p53 and tumour-specific.

### 2.4. ATR Inhibition Increases Radiosensitivity, in p53 Dysfunctional and wt Cells, but Not Nontumourigenic Immortalised Breast Cells

**Figure 3:**
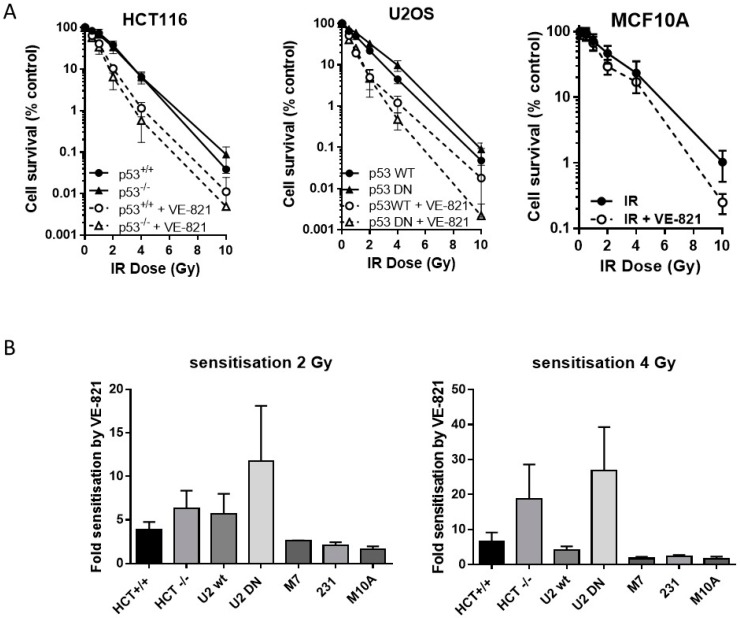
ATR inhibition increases radiosensitivity, irrespective of p53 status. (**A**) Survival of HCT116, U2OS or MCF10A cells exposed to increasing doses of irradiation in the presence of 1 µM VE-821 (open circles, dashed line) or a vehicle control of 0.1% DMSO (filled circles, solid line) for 24 h, then reseeded at low density for colony formation. Data are mean ± standard deviation from three separate experiments; (**B**) the surviving fraction at 2 Gy and 4 Gy was determined for paired and unpaired cell lines and the fold-sensitisation by VE-821 calculated from the ratio of the survival in the presence and absence of VE-821 for each independent experiment. HCT = HCT116, U2 = U2OS, M7 = MCF7, 231 = MDA-MB231, and M10A = MCF10A.

Neither the HCT116 p53^−/−^ nor the U2OS p53DN cells were more radioresistant than their p53 wt counterparts. VE-821 significantly increased the radiosensitivity of both pairs of cells (Figure 3A). We have shown previously [14] that VE-821 significantly potentiated IR in human breast cancer MCF7 cells (p53 wt) and in MDA-MB-231 cells (p53 mutant). Here we demonstrate that VE-821 did not increase the radiosensitivity of immortalised noncancer human breast MCF10A cells at the LC_50_ and all doses except for 10 Gy (Figure 3A). In HCT116 cells, p53 status had no significant effect on the extent of radiopotentiation by VE-821 at the LC_50_, but the sensitisation at 2 Gy and 4 Gy was 1.6 and 2.8-fold higher in the mutant cells, respectively (Figure 3B, Supplementary Table S4). In U2OS cells, VE-821 caused a significantly greater potentiation of p53 DN cells at the LC_50_ (*p* = 0.03) and sensitisation at 2 and 4 Gy was 2-fold and 6.5-fold greater in the mutant compared to wt. The p53 status of the breast cancer cell lines did not appear to affect the degree of potentiation of IR in each cell line, which was around 2-fold in both MCF7 cells and in MDA-MB-231 cells.

**Figure 4:**
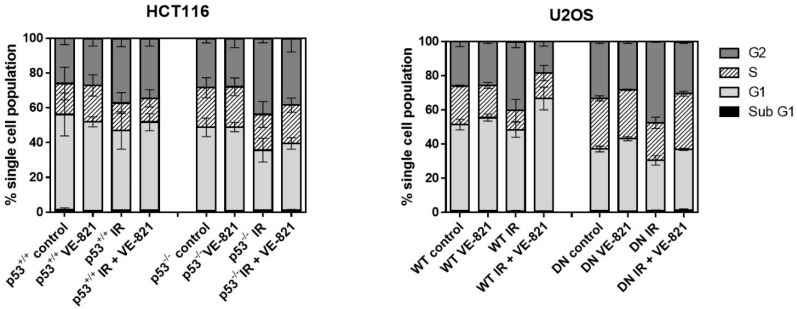
Effect of irradiation and VE-821 on cell cycle distribution of p53 wt and defective cells: Exponentially growing cells treated (where indicated) with 1 µM VE-821 for 1 h before IR (2 Grays) or mock treatment and the cell cycle distribution determined 24 h later. (Data are mean ± S.D. of three individual experiments).

As both p53 and ATR are implicated in different cell cycle checkpoints, it is likely that any radiopotentiation by VE-821 may be linked to changes in cell cycle profiles in each cell line. To establish whether any differences in radiopotentiation were due to cell cycle effects, each cell line was treated with a clinically relevant (2 Gy) dose of IR with or without VE-821. Consistent with mutated/deleted p53, untreated HCT116 p53^−/−^ and U2OS p53 DN cells had a reduced portion of G1 cells and a greater portion of S and G2 cells compared to their p53 wild-type equivalent cells (Figure 4). IR induced a substantial (43–57%) and significant (*p* < 0.05) increase in G2 arrest in all 4 cell lines, which did not appear to be p53-dependent, as contrasting results were observed in the p53 dysfunctional HCT116 and U2OS cells compared to their wt counterparts. VE-821 alone had negligible effects on cell cycle distribution but it reduced the IR-induced G2 arrest to a greater extent in the p53^−/−^ HCT116 cells (36%) than in the p53^+/+^ cells (24%) and completely abrogated the IR-induced G2 arrest in both of the U2OS cells.

## 3. Discussion

Four ATR inhibitors are now in clinical trial as monotherapy and/or in combination with DNA-damaging chemotherapy and ionising radiation. Results from a Phase I study of VX-970 (M6620) in combination with topotecan have recently been published along with other studies in abstract form [15,16,17]. These indicate that the drug is well tolerated as a single agent and in combination. In the study with topotecan, preliminary evidence was provided for inhibition of ATR and enhanced DNA damage in response to treatment with VX-970. Encouragingly, three of the five patients in the study that had small-cell lung cancer either had a partial response or experienced prolonged stable disease. Notably, small-cell lung cancer has a very high incidence of TP53 mutation at about 80%. Additional Ph1 studies are ongoing that will assess the efficacy of VX-970 in combination with various DNA damaging chemotherapies in TP53 mutant patients or in disease with a high rate of TP53 mutation, e.g., triple-negative breast cancer with a basaloid histology.

We set out to test the hypothesis that cells defective in p53 signalling would (a) be more sensitive to ATR inhibition by VE-821 as a single agent and (b) that VE-821 would chemo- and radio-sensitise p53 defective cells to a greater extent than wild-type cells, as suggested from previously reported work (reviewed in [1]). Radiosensitisation in HCT116 and U2OS cells has been reported previously [18] but the effect of p53 status on radiosensitisation in these cells was not investigated. In our previous study, we showed that radiosensitisation by VE-821 in unmatched breast cancer cell lines, MCF7 and MDA-MB-231, was similar [14]. Studies with CHK1 inhibitors have highlighted the difficulty in interpreting data using unmatched cell lines. For example, quite different levels of radiosensitisation were seen in 2 unmatched p53 mutant cell lines [19]. Similarly, potentiation of various chemotherapy drugs including gemcitabine by VX-970 was not significantly different in unmatched p53 wt and mutant cell lines [10]. Here, we used both isogenically matched cells and the nonmatched breast cancer cells. We determined cytotoxicity directly by the ability to form colonies of >30 cells, which we believe is the most reliable method as indirect measures have been shown to give contradictory results [20], indeed our own data have shown that, due to the cytostatic effects of VE-821, proliferation assays are not always representative of longer-term cytotoxicity [21].

Inhibition of ATR activity varied between the cell lines but there was considerable interassay variability as Western blotting can only be considered to be semiquantitative. Nevertheless, in the multiple experiments performed there was no indication that p53 status had any impact on the ability of VE-821 to inhibit ATR intracellularly.

ATR inhibitors have been shown to cause elevated levels of replication stress in p53-defective cells [22] and previously published data indicated that ATR knockdown reduced clonogenic survival in p53 null HCT116 cells compared to those with wt p53 [23]. However, we found that dysfunctional p53 did not increase the sensitivity of cells to VE-821 as a single agent. These data suggest that endogenous replication stress is insufficient to create the dependence on ATR signaling, and hence sensitivity to ATR inhibition, in the absence of p53-dependent G1 checkpoint signaling. One possibility is that the loss of p53 renders the cells less likely to undergo apoptosis but there was no increase in sub-G1 cells detected after 24 h exposure to VE-821. Further studies focused on a variety of apoptosis measurements would be needed to confirm this hypothesis given the limitations of measurement of apoptosis by subG1 peak. It should be noted, however, that loss of G1 checkpoint control is common in cancer [24] and that many cell lines commonly considered to have wt p53 have other downstream defects that render them incapable of mounting a functional p53 response [25]. HCT116 have hemimethylated p16 [26] and fail to increase p21 in response to topoiosomerase I poisoning (SN38) [25]. In U2OS cells, neither p16 (INK4A) nor p14 (ARF) are expressed due to promoter hypermethylation [27]. Indeed, one of the earliest investigations of ATR as a target indicated a variety of factors affecting G1-S transition increased the sensitivity of cells to ATR inhibition [28]. Certainly, our cell cycle analysis indicated that none of the cancer cells arrested in G1 after irradiation irrespective of p53 status.

Previously published data indicated that CHK1 inhibition did not cause any differential radiosensitisation between p53 wt and null HCT116 cells [29]. In contrast, we found that radiosensitisation by VE-821 was greater in the p53 null and mutant cells compared to p53 wt HCT116 and U2OS cells. This cannot be attributed to different methodology because the previous report also used clonogenic survival and, therefore, this may indicate an important difference between ATR and CHK1 inhibition. In support of this hypothesis, early data using ATR-kinase dead activation in U2OS cells identified greater radiopotentiation when p53 function was impaired by MDM2 or HPV-E6 transduction [30]. Radiosensitisation by VE-821 was more marked at higher doses, suggesting that there is an increased dependence on ATR signaling with increasing levels of DNA damage-induced replication stress. Such higher doses are more representative of hypofractionated radiotherapy. There is considerable interest in hypofractionated radiotherapy, by external beam and stereotactic body radiotherapy or proton therapy, currently. Along with the obvious greater convenience and cost benefits of fewer hospital visits hypofractionation provides, in many cases it appears to be equally beneficial and associated with less severe acute and short-term toxicities in breast (2.5 Gy/fraction) and head and neck cancer (6 Gy/fraction) [31,32,33]. Moreover, meta-analysis revealed that moderate hypofractionation (2.5–4 Gy/fraction) and accelerated hypofractionation (5–10 Gy/fraction) was associated with improved biochemical control in prostate cancer [34]. It is tempting to speculate that the lack of radiosensitisation in the noncancer MCF10A cells at doses <10 Gy suggests that the combination of ATR inhibition with moderately hypofractionated radiation therapy would achieve tumour-selective radiosensitisation and be well tolerated.

Gemcitabine is incorporated into DNA causing chain termination and also inhibits ribonucleotide reductase that limits the production of dNTPs, both of which cause profound replication stress [35] and activate checkpoint signalling [36]. It is not surprising, therefore, that very substantial chemosensitisation by VE-821 of gemcitabine cytotoxicity was observed in all cell lines. This was particularly striking at the higher concentrations of gemcitabine. Previous studies had indicated that p53 status was not a determinant of chemosensitisation of other antimetabolites (methotrexate, 5FU) by CHK1 knockdown or inhibition by UCN01 in U2OS cells [37]; however, in these studies methylene blue was the read-out of cell viability, rather than clonogenic survival as we have done. In contrast, studies in A549 lung cancer cells demonstrated that p53 knockdown caused an approximately 2-fold increase in the chemosensitisation of gemcitabine by VX-970 [10]. We found that at 100 nM gemcitabine VE-821 reduced the clonogenic survival of the p53 dysfunctional HCT116 cells and U2OS cells by around 60-fold and 35-fold, respectively, and 16-fold in the MDA-MB231 cells compared with 2-fold and 2.3-fold in the p53 wt HCT116 and U2OS cells, respectively, and 4-fold in the MCF7 cells. Such levels of gemcitabine are easily achievable as with prolonged infusions of 10 mg/m^2^/min over 8 h the plasma steady-state levels are reported to be between 10 and 30 µM [38,39]. Encouragingly, there was no sensitisation of gemcitabine in the nontumourigenic MCF10A cells and thus we are tempted to predict that chemosensitisation will be tumour-specific and that it may be possible to lower the dose of gemcitabine when given in combination with ATR inhibitors with the same level of tumour control and less toxicity.

Good levels of chemo- and radiosensitisation by VE-821 were also achieved in the p53 wt cells with >4-fold sensitisation at all doses of IR and most concentrations of gemcitabine. This clearly suggests that some cells can respond to ATR inhibition even in the absence of p53 loss of function. Given that noncancer cells, MCF-10A, as reported here, and MCF5 and HFL-1 as reported elsewhere [40], are robustly insensitive to ATR inhibitor gemcitabine and irradiation sensitisation, some event in these cancer cells must be rendering them sensitive. Accordingly, additional studies to define further general markers of response to ATR inhibition are merited. Furthermore, in the chemo- and radiosensitisation studies the combination data was normalised to VE-821 alone and it should be remembered that at 1 µM VE-821 caused around 50% reduction in survival in the p53 dysfunctional cells vs. 17% in the p53 wt HCT116 cells and 34% in U20S so, although the chemo and radiosensitisation by VE-821 was greater in the p53 dysfunctional cells, the combined cytotoxicity of the 2 drugs was more similar. In the unmatched breast cancer cells, sensitisation was greater in the p53 mutant MDA-MB-231 cells despite the cytotoxicity of VE-821 alone being similar in both cell lines.

In summary, we believe that our data indicate that loss of G1 checkpoint control is a determinant of chemo- and radiosensitisation by ATR inhibition, but that p53 status is just one factor contributing to the effect. Most encouragingly, neither radiosensitisation nor chemosensitisation was significant in the nontumourigenic breast MCF10A cells, suggesting that sensitisation is tumour-specific. The greater sensitisation of higher doses of radiation suggest that the combination with hypofractionated radiotherapy may produce the best results. Additionally, the substantial gemcitabine potentiation, at concentrations well within achievable clinical concentrations, suggest that it may be possible to combine less intensive gemcitabine therapy with ATR inhibition and achieve similar antitumour activity with reduced toxicity. Our finding that the cytotoxicity of ionising radiation and gemcitabine are both substantially enhanced by VE-821 in breast, colon, and osteosarcoma cells are in good agreement with studies in pancreatic cancer [40]. Furthermore, gemcitabine in combination with radiotherapy has shown clinical benefit in lung and pancreatic cancer [41,42] and these data suggests that addition of an ATR inhibitor to such trials may prove beneficial in several types of cancer independently of p53 status.

## 4. Methods

### 4.1. Chemicals and Reagents 

Routine chemicals and reagents were obtained from Sigma Aldrich (Poole, UK) unless otherwise stated. The ATR inhibitor VE-821 (Vertex Pharmaceuticals (Europe) Ltd., Abingdon, UK) was dissolved in DMSO and stored at −20 °C.

### 4.2. Cell Lines 

All cell lines used were obtained from the Northern Institute for Cancer Research bank of authenticated (using short tandem repeat DNA profiling: LGC Standards) cells. Postauthentication passages were limited to 30 (<6 months) before replacing with a lower passage. Cells were confirmed as free from mycoplasma (Mycoalert, Lonza Group Ltd., Basel, Switzerland) every 6–8 weeks. MCF7 (p53 wt), MDA-MB-231 (p53 mutant) breast cancer cells and HCT116 human colorectal carcinoma cells, either p53 wild type (HCT116 p53^+/+^) or p53 deleted (HCT116 p53^−/−^) were cultivated in RPMI1640 medium supplemented with 10% foetal bovine serum. MCF10A immortalised human breast epithelial cells and U2OS human osteosarcoma cells with wild-type p53 (U2OS p53 WT) were maintained in DMEM with 10% foetal bovine serum. U2OS cells overexpressing the R175H variant of p53 that is reported to have a dominant negative effect (U2OS p53 DN) [43] were grown in the presence of 400 µg/mL G418 to select for cells expressing the dominant negative construct.

### 4.3. ATR Inhibition

Exponentially growing cells in duplicate wells of a 6-well plate were co-exposed to 1 µM gemcitabine, to activate ATR, with increasing concentrations of VE-821 for 1 h. 10 mM HU was used as a positive control as it is known to activate ATR [9]. VE-821 stock was diluted in DMSO at 200× the final concentration before diluting 1 in 200 in medium and 0.5% DMSO was used as a vehicle control. Lysates were run on 18-well Criterion gels to allow multiple samples to be run at the same time (Bio-Rad, Watford, UK). ATR activity was measured by CHK1 phosphorylation by Western blotting. Antibodies to pCHK1^Ser345^ (rabbit 133D3, 1/300 Cell Signaling, Danvers, MA, USA), ATR (goat N-19, 1/300, Santa Cruz Biotechnology, Dallas, TX, USA) and actin (mouse AC-40, 1/1000, Sigma Aldrich) and the appropriate HRP-conjugated secondary antibodies (antigoat HRP, 1/2000–Santa Cruz Biotechnology, Dallas, TX, USA, antirabbit HRP 1/1000 and antimouse HRP (1/2000–Dako UK Ltd., Ely, UK) were used. Protein expression was measured by chemiluminescence from exposure to ECL Prime detection reagent (GE Healthcare, Pittsburg, PA, USA) using a G-box (Syngene, Cambridge, UK). Densitometry of bands was carried out using Genetools software (Syngene, Cambridge, UK).

### 4.4. Cytotoxicity

Exponentially growing were either exposed to increasing concentrations of VE-821 alone for 24 h, or irradiated or treated with gemcitabine in the presence or absence of 1 µM VE-821 for 24 h, then harvested and seeded for colony formation in drug-free medium. Where cells were exposed to IR and VE-821, cells were treated for 1 h with 1 µM VE-821 before irradiation. Depending on the growth rate of the cells 1–2 weeks later colonies were fixed with methanol and visualised with crystal violet and counted.

### 4.5. Cell Cycle Analysis

Exponentially growing cells were seeded into 10 cm tissue culture plates (1 million cells per dish) and allowed to adhere for 24 h. They were then exposed to 2 Gy irradiation or mock-irradiated in the presence or absence of 1 µM VE-821 for 24 h. Following treatment, the cellular media was collected and the cell washed in PBS. The PBS used for washing was also collected and added to media previously collected. Cells were harvested using trypsin and resuspended in full medium and added to previously collected media and PBS. Cells were pelleted washed in PBS, pelleted again and carefully resuspended in ice cold methanol to fix the cells. Cells were then stored for a minimum of 12 h at −20 °C. Cells were pelleted-washed twice in PBS then resuspended in PBS containing 200 µg/mL propidium iodide to stain the DNA and 200 µg/mL RNAase A to degrade any RNA that may interfere with detection of DNA. Cells were incubated in the dark for 30 min at room temperature before being run on a BD FACSCalibur (BD Biosciences, San Jose, CA, USA) at a rate of approximately 12 µL/minute to detect propidium iodide DNA staining and model a cell cycle profile. Cell cycle profiles were quantified using Cyflogic software (CyFlo Ltd., Turku, Finland).

## 5. Conclusions

Defective p53, whilst not conferring greater sensitivity to ATR inhibition by single agent VE-821 consistently conferred greater chemo- and radiosensitisation, in isogenic cells but not in unmatched cells. We therefore conclude that p53 status is just one factor contributing to chemo- and radiosensitisation by ATR inhibition. The lack of chemo- or radiosensitisation in the noncancerous MCF10A cells suggests an element of tumour-specificity that warrants further investigation. The greater sensitisation at high-dose irradiation suggests that ATR inhibitors may be most effective with hypofractionated radiotherapy.

## Supplementary Materials

The following are available online at http://www.mdpi.com/2072-6694/10/8/275/s1, Table S1: ATR inhibition by VE-821 in the cell line panel. Table S2: VE-821 single-agent cytotoxicity. Table S3: Summarised chemosensitisation data for all cell lines. Table S4: Summarised Radiosensitisation by VE-821 in all cell lines.
