# Supplementary material for: The Impact of p53 Dysfunction in ATR Inhibitor Cytotoxicity and Chemo- and Radiosensitisation
Source: Cancers (Basel). 2018 Aug 20;10(8):275. doi: 10.3390/cancers10080275 (PMC6116113; doi:10.3390/cancers10080275)
Supplement: Supplementary file 1 [file cancers-10-00275-s001.pdf]

Supplementary Materials: The Impact of p53 Dysfunction in ATR Inhibitor Cytotoxicity and Chemo- and Radiosensitisation

Fiona K. Middleton, John R. Pollard and Nicola J. Curtin

Table S1. ATR inhibition by VE-821 in the cell line panel. Cells were co-exposed to 1 μM gemcitabine with increasing concentrations of VE-821 for 1 hour. The IC_{50} was interpolated from concentration–response curves. Data are mean and SEM of 3 independent experiments or individual measurements where only 2 experiments were valid.

**p53 wt**		**p53 mutant**	
Cell line	IC_{50} (μM)	Cell line	IC_{50} (μM)
HCT116	0.92 ± 0.34	HCT116 p53−/−	2.46 ± 0.42
U20S	8.95, 4.5	U2OS p53DN	4.85 ± 1.77
MCF7	3.62 ± 1.94	MDA-MB-231	0.57 ± 0.30
MCF10A	1.16 ± 1.32		

Table S2. VE821 single-agent cytotoxicity. Pooled LC_{50} values interpolated from survival curves from ≥3 individual experiments as shown in Figure 1B. Data are mean ± SEM from values calculated for each individual experiment. Significance of difference between p53 wt and mutant/null is given in parenthesis.

**p53 wt**		**p53 mutant**	
**Cell line**	**LC_{50} (μM)**	**Cell line**	**LC_{50} (μM)**
HCT116	2.13 ± 1.0	HCT116 p53−/−	4.56 ± 1.79 ($p = 0.1$)
U20S	2.54 ± 2.21	U2OS p53DN	3.34 ± 2.74 ($p = 0.7$)
MCF7	1.89 ± 0.81	MDA-MB-231	1.93 ± 0.93 ($p = 1$)
MCF10A	>10		

Table S3. Summarised chemosensitisation data for all cell lines. LC_{50} values were calculated and compared within each cell line and the potentiation factor at 50% (PF_{50}) cell kill compared between cell lines; both cancer cell lines were potentiated >4-fold but there was no potentiation in MCF10A (i.e. $PF_{50} < 1$).

**Cell line**	**LC_{50} gemcitabine) (nM)**	**LC_{50} gemcitabine +VE-821 (nM)**	**Fold potentiation by 1 μM VE-821**
MCF10A	7.8 ± 1.8	10.0 ± 5.4	0.87 ± 0.13
MCF7	53.4 ± 3.0	12.7 ± 5.3	4.9 ± 1.46
MDA-MB-231	54.6 ± 29.1	16.5 ± 11.0	4.3 ± 1.5
HCT116	24.6 ± 31.0	11.5 ± 6.1	1.5 ± 0.9
HCT116 p53−/−	42.0 ± 36.2	8.7 ± 2.1	4.3 ± 1.4 ($p = 0.035$)
U2OS	13.7 ± 5.7	6.9 ± 0.5	1.97 ± 0.44
U2OS DNp53	11.1 ± 7.3	6.0 ± 0.6	1.76 ± 0.58

Table S4. Summarised Radiosensitisation by VE821 in all cell lines. Data are mean ± SEM of 3 independent experiments, p values are from unpaired *t*-tests of the values obtained in each independent experiment. (SF = surviving fraction (%))

Treatment	HCT116+/+	HCT116-/-	U2OS wt	U2OS DN	MCF7	MDA-MB-231	MCF10A
SF 2Gy	37.7 ± 5.3	33.3 ± 5.6	21.9 ± 1.7	32.6 ± 2.3	31.2 ± 6.4	33.1 ± 4	46.7 ± 7.2
SF 2Gy + VE-821	10.3 ± 1.3	6.4 ± 1.9	4.9 ± 1.4	4.7 ± 1.7	12.0 ± 2.6	16.7 ± 1.8	30.4 ± 4
fold-sensitization	3.9 ± 0.9	6.4 ± 2.01	5.72 ± 2.27	11.7 ± 6.4	2.6 ± 0.04	2.05 ± 0.39	1.61 ± 0.34
p	0.0074	0.011	0.002	0.0006	0.05	0.020	0.118
SF 4Gy (%)	6.44 ± 1.2	6.5 ± 0.89	4.42 ± 0.55	9.71 ± 1.6	3.78 ± 0.76	13.7 ± 1.95	23.4 ± 6.8
SF Gy + VE-821 (%)	1.13 ± 0.25	0.57 ± 0.23	1.2 ± 0.3	0.47 ± 0.12	2.32 ± 0.33	6.03 ± 0.78	20.6 ± 12
fold-sensitization	6.67 ± 2.5	18.7 ± 9.9	4.11 ± 1.08	26.8 ± 12.5	1.74 ± 0.45	2.32 ± 0.36	1.67 ± 0.59
p	0.013	0.003	0.007	0.005	0.152	0.022	0.851

© 2018 by the authors. Submitted for possible open access publication under the terms and conditions of the Creative Commons Attribution (CC BY) license (http://creativecommons.org/licenses/by/4.0/).
